# Preemptive Oral Etoricoxib on Health-Related Quality of Life after Mandibular Third Molar Surgery: A Randomized, Double-Blind, Placebo-Controlled Clinical Trial

**DOI:** 10.1155/2021/8888151

**Published:** 2021-03-06

**Authors:** Long Xie, Zhi Li, Zheng-Jun Shang

**Affiliations:** ^1^The State Key Laboratory Breeding Base of Basic Science of Stomatology (Hubei-MOST) & Key Laboratory of Oral Biomedicine Ministry of Education, School & Hospital of Stomatology, Wuhan University, Wuhan, China; ^2^Department of Oral and Maxillofacial Surgery, School and Hospital of Stomatology, Wuhan University, Wuhan, China

## Abstract

This study was aimed at evaluating the use of oral etoricoxib for preemptive analgesia on the health-related quality of life (QoL) outcome after the extraction of mandibular third molar. The study population consisted of 60 participants that required extraction of a single partial bony impacted mandibular third molar under local anesthesia and met the inclusion criteria. The participants were randomized into two groups. The etoricoxib group orally received 60 mg etoricoxib 30 min before surgery, whereas the control group was given a placebo. The patients were assessed postoperatively after 1, 2, 3, 4, 5, 6, and 7 days using the United Kingdom oral health-related QoL questionnaire and visual analog scale for maximum postoperative pain. The total dose of ibuprofen rescue intake and total number of days the drug was taken were recorded. Surgical removal of impacted teeth had a negative influence on the patient's QoL across various physical, social, and psychological aspects. The scores for postoperative pain in the etoricoxib group were significantly lower than those in the control group on each postoperative observation day. The number of patients without analgesic rescue medication, the average amount, and total number of days emergency analgesics were taken were significantly lower in the etoricoxib group than in the control group. The etoricoxib group showed better QoL score than the control group. Preemptive oral etoricoxib is an effective therapeutic strategy for improving the QoL after surgical removal of the impacted lower third molar.

## 1. Introduction

The extraction of impacted mandibular third molars under local anesthesia involves bone and muscle tissue damage, which results in inflammatory complications, such as pain, swelling, trismus, and alveolitis, in the immediate postoperative period [1]. These complications may significantly lead to deterioration in the quality of life (QoL) during the immediate postoperative period [2]. Various drug treatment methods have been employed and compared to solve this problem. Preemptive oral etoricoxib is an important factor affecting the frequency and severity of postoperative complications [3–7].

Preemptive approaches focus on preventing postoperative algesic flare and moderation or blockage of the occurrence of hyperalgesic states. Preemptive analgesic strategies are used to control or prevent central sensitization [8]. Preemptive oral etoricoxib (120 mg) is effective in providing analgesia after total abdominal hysterectomy, single-level discectomy, and inguinal hernia repair [9–11].

COX-2 inhibitors have been shown to have more analgesic effects than ibuprofen after impacted tooth extraction [12]. Etoricoxib with powerful analgesic effects is a new orally active cyclooxygenase enzyme- (COX-) 2 inhibitor. This compound has a 100-fold higher selectivity for the COX-2 receptor than COX-1 and has the added convenience of once-a-day dosing [13].

QoL can be defined as the patient's subjective perception of the effects of a disease and its treatment on their daily life, physical, psychological, and social functioning and well-being. In one study, the highest negative effect of third molar surgery on the QoL of patients on the first postoperative day (POD) decreased over the follow-up period [2]. In addition, the teeth considered associated with technical difficulties for extraction on the basis of their position have low health-related QoL score [14]. The United Kingdom oral health-related QoL (UK-OHRQoL, (based on the WHO's “structure-function-ability-engagement” model) questionnaire can be used to measure the QoL sensation of patients after third molar removal [15].

To the best of our knowledge, this research is the first to assess the effect of preemptive oral etoricoxib application on surgical removal of impacted mandibular third molars on a patient's postoperative QoL.

## 2. Materials and Methods

### 2.1. Patients and Methods

The study was performed in accordance with the Declaration of Helsinki regarding medical research. Ethical approval was obtained at the beginning of the study from the Ethics Committee of School & Hospital of Stomatology, Wuhan University (approval number 2019-B11). This study was registered at clinicaltrials.gov (Clinical Trials Registry No. ChiCTR1900024503).

The study consisted of 60 participants, with 31 (51.7%) females and 29 (48.3%) males. The exclusion criteria were the presence of systemic diseases, development of local infections, smoking, pregnancy, currently breastfeeding, and use of contraception. All participants were patients at the Department of Oral and Maxillofacial Surgery of Hospital of Stomatology of Wuhan University and with complete indications for removal of the impacted third molar. The participants were given standardized participant information sheets, and they provided written informed consent to join the study. All patients were informed about the study protocol and possible risks prior to any procedure.

**Figure 1:**
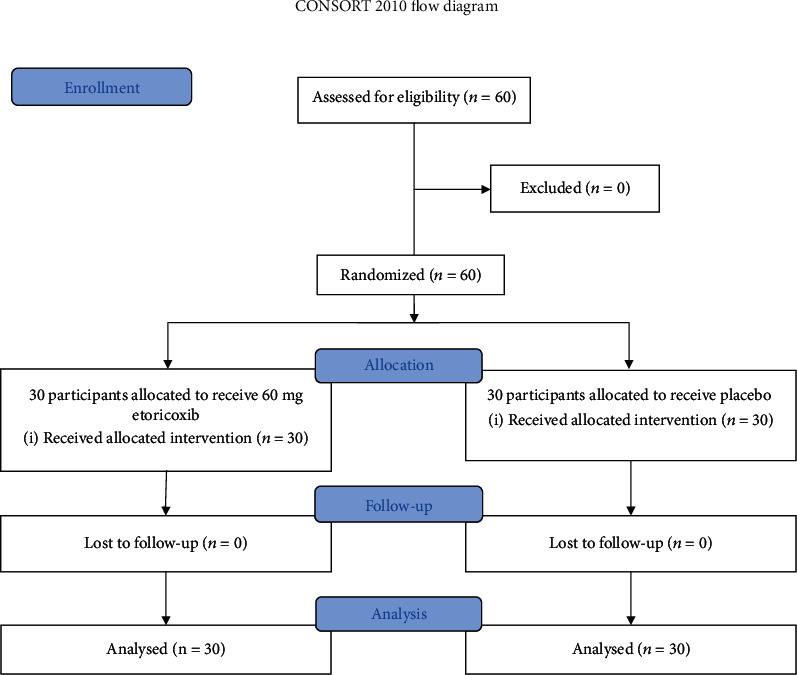
Patient enrollment flow diagram.

Sixty patients were randomly divided into two treatment groups by using a series of random numbers, with each group comprising 30 patients (Figure 1). The etoricoxib group orally received 60 mg etoricoxib, whereas the control group was orally administered with placebo. These treatments with preemptive analgesia were performed 30 min before the surgery. The patient and the operator were blinded to the type of drugs administered.

### 2.2. Calculation of Sample Size

A pilot study was conducted on 16 patients (8 patients for each group) to assist in the calculation of sample size for the main study to enable statistical rejection of the null hypothesis with an 80% power and 95% confidence interval. A group sample size of 30 achieved 80% power with a significance level (alpha) of 0.05 using a two-sided two-sample *t*-test.

### 2.3. Surgical Procedure

All operations were performed by the same oral surgeon to minimize differences between operators. The same technique for application of local anesthesia was performed on the patients. In particular, 2% lidocaine was used to block the inferior alveolar, lingual, and long buccal nerves. Local infiltration anesthesia was achieved using 4% artecaine and 1 : 100,000 epinephrine (Septanest, Septodont, France). Both groups underwent the same surgical procedure to reduce surgery-related bias. The same triangular flap design was used for all gingival flaps. Buccal surgical approach was performed with the incision of mucoperiosteal flap. The 45° elevation high-speed turbine mobile phone was used to remove the bone and cut the crown; tooth removal was performed using elevating instruments in the appropriate direction. After tooth extraction, the alveolar tissue was scraped and rinsed with sterile saline. The wound was sutured with a 4-0 silk, and the suture was removed 1 week after the surgery.

### 2.4. UK-OHRQoL Evaluation Criteria

After the surgery, each patient was given a diary, which is a condition-specific 16-item UK-OHRQoL measure [16]. Each patient was instructed to complete the diary each postsurgery day for 7 days. The OHRQoL instrument is designed to assess a patient's perception of recovery in three main categories, namely, physical, psychological, and social aspects, which are related to the removal of third molars. Each item was scored as follows: none, 0; little, 1; moderate, 2; great, 3; and extreme, 4. The total scores ranged from 0 to 64. A high score indicates poor QoL. The domain scores are presented in Results.

### 2.5. Pain Assessment

An 11-point (0–10) visual analogue score (VAS) was used to assess pain (0, no pain; 1–3, mild pain; 4–6, moderate pain; 7–9, severe pain; 10, worst possible pain). The maximum postoperative pain was scored using the VAS scale of 1, 2, 3, 4, 5, 6, and 7 days after surgery. Ibuprofen (300 mg) was prescribed as the rescue drug only in the case of VAS > 3. The patients also jotted daily notes of their total ibuprofen consumption within 1 week. At the end of the 1-week recovery period, the patients were asked to return the completed diary to the data center.

### 2.6. Statistical Analyses

Data were analyzed using SPSS software (SPSS, Inc., USA), and independent *t*-test and *χ*^2^ test were used to determine the significant difference between groups. Parametric outcomes were expressed as the mean ± standard deviation (SD).

## 3. Results

The mean age of patients (29 men and 31 women) was 28.5 ± 5.0 years (range: 18–48 years). No statistically significant differences were found in the demographic and clinical characteristics among the study groups (Table 1). No data were missing, and all patients attended all study visits. No cases of alveolar osteitis nor wound infection were reported during follow-up. No side effects of the drugs used in the trial were mentioned nor noted (Table 1).

Table 2 shows that the mean VAS for pain in the etoricoxib group was lower than that in the control group at all intervals (*p* < 0.05).

Administration of etoricoxib before the surgery significantly reduced the consumption of prescribed medication (amount of tablets, mean ± SD; Table 3). During the 1-week period, the etoricoxib group received an average of 1.3 ± 2.0 prescription dose, whereas the control group took a prescription dose of 4.2 ± 4.2 (*p* = 0.002). In the etoricoxib group, 53.3% of the patients did not take any postoperative analgesic, and this number was 43.3% higher than that in the control group (*p* < 0.001). The total number of days emergency analgesics were taken was 0.8 ± 1.1 in the etoricoxib group, and it was less than that in the control group (2.4 ± 2.0).

The mean total and subscale scores were the highest among all groups on the first POD; the values gradually improved throughout the immediate postoperative period.

The etoricoxib group showed statistically significant differences in the total QoL during the immediate postoperative period. This group also showed a significant difference in the effect of QoL in terms of the physical and psychological aspects (*p* < 0.05) at PODs 1–7 and social aspect at PODs 2–6 compared with the control group (Table 4).

## 4. Discussion

This study was aimed at evaluating the effect of preemptive oral etoricoxib on postoperative pain and QoL measures after third molar surgery. We hypothesized that preemptive oral etoricoxib has a significant positive effect on the QoL of patients. The preemptive oral administration of etoricoxib can improve the QoL after third molar surgery.

Etoricoxib is a second-generation selective class of nonsteroidal anti-inflammatory drug approved for the treatment of patients with rheumatism and osteoarthritis. Etoricoxib is chemically written as [5-chloro-2-(6-methylpyridin-3-yl)-3-(4- methylsulfonylphenyl) pyridine]. This compound is a dipyridinyl derivative that has a (4-methylsulfonyl) phenyl group attached to the central ring [17]. Etoricoxib has more than 100-fold selectivity for COX-2 versus COX-1 in various cell and whole-blood assays. This agent is less active against COX-1 than other selective COX-2 inhibitors and offers the major advantage of reduced gastrointestinal toxicity [13, 18].

Etoricoxib is an effective preemptive analgesic for patients undergoing total abdominal hysterectomy, single-level discectomy, and inguinal hernia repair [10, 11, 13]. Oral etoricoxib is rapidly and completely absorbed. Etoricoxib has a marked distribution in tissues (distribution volume of 119 L) and is 92% bound to plasma proteins. This chemical distributes rapidly, reaching its peak concentration within 1–2 h. Etoricoxib has an elimination half-life of approximately 22 h [19].

Evidence suggests that certain treatments can improve the QoL after tooth extraction. Majid confirmed that submucosal injection of dexamethasone (4 mg) can greatly improve the QoL [20]. Batinjan et al. observed that QoL can be ameliorated by laser therapy [21]. However, no study assessed the effect of etoricoxib on the QoL after third molar surgery. Two studies investigated the QoL of orthopedic patients on the basis of etoricoxib use. Ramos-Remus et al. demonstrated that the use of etoricoxib on patients with osteoarthritis, rheumatoid arthritis, or chronic low-back pain is associated with improvements in all Short Form-8 Health Survey QoL domains and component scores and the measures of pain and physical functioning [22]. However, Eichler et al. insisted that the degree of association between the changes in QoL variables is low except for bodily pain [23]. Overall, whether etoricoxib can improve QoL is unclear.

In this study, the etoricoxib group showed statistically significant differences in the total QoL in the immediate postoperative period. The extraction of impacted teeth had a negative effect on the QoL in all aspects of the patient's life, including restrictions in daily life activities, e.g., the ability to chew food, open mouth, speak, comfort, laugh, and sleep.

Third molar surgery in the control group was associated with the deterioration of QoL in the immediate postoperative period [10, 21, 24]. This finding was reflected in the high scores of all the QoL subscales recorded in the questionnaire.

Preemptive etoricoxib had a significantly positive effect on the physical aspects of the QoL of patients, including enjoyment of food, appearance, speech, general health, comfort, and breath odor. The score for enjoyment of food was lower in the etoricoxib group than in the control group on PODs 1–6. According to Shugars et al., Oral Health Impact Profile-14 responses suggest that pain affects the ability to eat [25]. In the present study, the pain reported in the etoricoxib group at a level less than the control group resulted in enhanced ability to eat at PODs 1–6. Albuquerque et al. pointed out that preemptive etoricoxib leads to a significant reduction in tumor necrosis factor-*α* concentration, resulting in significantly reduced clinical parameters of pain, trismus, and edema compared with the placebo group [4]. The low influence on speech in the etoricoxib group is possibly due to the anti-inflammatory effect of etoricoxib. Severe trismus in the control group prevented patients from eating normally and brushing their teeth, affecting their general health and breath odor. Enhanced physical aspects are related to preemptive analgesia.

In this study, tooth extraction surgery affected the daily social life of patients at PODs 2–6. The patients in the etoricoxib group can smile more and perform more daily work than those in the control group. Sancho-Puchades et al. removed all four third molars from outpatients under conscious sedation, which caused patients to stop working for an average of 4.9 days [26]. The reduction in the degree of impairment of social activities in the etoricoxib group may be due to the anti-inflammatory effect of etoricoxib. Savin and Ogden considered that a major problem with socialization is related to altered facial appearance [27].

With regard to psychological aspects, preemptive analgesia can improve the results in patients who underwent impacted tooth extraction. Sleep disturbances may be attributed to sleep disruptions caused by the discomfort related to tooth extraction and drowsiness caused by postoperative medication [28]. Ibikunle et al. reported that the proportion of sleep disorders among subjects who received prednisolone was significantly lower than that of those who did not receive prednisolone [29]. Poor sleep quality affects the patients' confidence, carefree manner, mood, and personality. The patients in the etoricoxib group performed better psychologically than those in the control group, because the former took less emergency analgesics and used them for a shorter period of time. In addition, the anti-inflammatory effect of etoricoxib affected these patients psychologically.

It was worth noting that the results of our study were different from those of Isola et al. [30], and the possible three reasons were as follows: the first was ethnic differences, the second was the difference in the technical proficiency of the operators, and the third and the most important reason was the difference in the time point of oral analgesics. Preoperative administration of etoricoxib can ensure stable concentration in key tissues, such as plasma, CSF, and wound fluid after surgery [31]. This process resulted in a complete blockade of PGE2 production in the surgical wound and CSF. This phenomenon can lead to pain relief and reduce demands for postoperative analgesic.

There were some limitations in this study. First, due to the development of the internet, it was easier for patients to obtain methods to improve the quality of life after surgery from the network, such physiotherapy as ice compress and chlorhexidine-based mouthwash [32, 33]. Second, dental anxiety was not assessed preoperatively, which was associated with pain and affects quality of life [34]. Third, the split-mouth randomized clinical trial design was not used in this study. The above three points may have some influence on this study.

This study indicated that preemptive etoricoxib caused a significant improvement in health-related QoL after surgical removal of impacted lower third molars.

## Figures and Tables

**Table 1:** Demographic data.

	Control	Etoricoxib	*p* value
Age (years)	27.5 ± 3.9	29.5 ± 5.8	0.109^*α*^
Weight (kg)	63.5 ± 17.0	66.5 ± 12.7	0.433^*α*^
Height (cm)	168.0 ± 7.1	170.4 ± 5.5	0.150^*α*^
BMI (kg/m^2^)	22.3 ± 4.6	22.8 ± 3.7	0.608^*α*^
Gender (M/F)	11/19	18/12	0.071^*β*^
Duration of the operation (minutes)	15.2 ± 3.9	16.1 ± 4.2	0.376^*α*^

BMI: body mass index. Note: data are presented as mean (standard deviation) or number. *^*α*^*By *t*-test. *^*β*^*By *χ*^2^ test.

**Table 2:** Average pain measurements in study groups.

Timing of VAS score, days	Control	Etoricoxib	*p* value
POD 1	5.6 ± 2.7	2.9 ± 2.2	*p* < 0.001^∗∗∗^
POD 2	4.7 ± 3.0	1.9 ± 1.9	*p* < 0.001^∗∗∗^
POD 3	3.7 ± 2.9	1.5 ± 1.7	0.001^∗∗^
POD 4	3.0 ± 2.7	0.8 ± 1.1	*p* < 0.001^∗∗∗^
POD 5	2.3 ± 2.3	0.5 ± 0.8	*p* < 0.001^∗∗∗^
POD 6	1.5 ± 1.8	0.4 ± 0.6	0.002^∗∗^
POD 7	1.0 ± 1.4	0.4 ± 0.7	0.04^∗^

Values are expressed mean ± standard deviation. VAS: visual analog pain scale. Values are expressed mean ± standard deviation or number. ^∗^*p* < 0.05*t*-test between groups. ^∗∗^*p* < 0.01*t*-test between groups. ^∗∗∗^*p* < 0.001*t*-test between groups.

**Table 3:** Comparison of the average dosage and number of patients during 7-day rescue analgesic intake among groups.

Variable	Control (*n* = 30)	Etoricoxib (*n* = 30)	*p* value
Number of patients who consumed the first rescue analgesic medication during the period of evaluation (7 d)	27 (90%)	14 (46.7%)	*p* < 0.001^*β*^
Number (%) of patients requiring no rescue analgesic medication during the period of evaluation (7 d)	3 (10%)	16 (53.3%)	*p* < 0.001^*β*^
Total analgesic consumption for postoperative 7 d (tablets) (mean ± SD)	4.2 ± 4.2	1.3 ± 2.0	0.002^*α*^
Total number of days taken with emergency analgesics	2.4 ± 2.0	0.8 ± 1.1	*p* < 0.001^*α*^

Note: data are presented as mean (standard deviation) or number. ^*α*^By *t*-test. ^*β*^By *χ*^2^ test.

**Table 4:** Distribution of mean quality of life score domains in patient groups at postoperative days, mean (SD).

Subscales of QOL	POD 1		POD 2		POD 3		POD 4		POD 5		POD 6		POD 7	
	Control	Etoricoxib	Control	Etoricoxib	Control	Etoricoxib	Control	Etoricoxib	Control	Etoricoxib	Control	Etoricoxib	Control	Etoricoxib
Eating/enjoyment of food	3.0 (1.1)	2.5 (0.8)^∗^	2.6 (1.1)	1.8 (0.8)^∗∗^	2.3 (1.1)	1.3 (0.9)^∗∗∗^	1.8 (1.1)	0.8 (0.6)^∗∗∗^	1.4 (1.1)	0.6 (0.7)^∗∗^	1.0 (1.0)	0.4 (0.5)^∗∗^	0.7 (0.9)	0.3 (0.5)
Appearance	1.4 (1.1)	1.2 (0.9)	2.0 (1.2)	1.3 (1.0)^∗^	2.0 (1.2)	1.1 (1.2)^∗^	1.6 (1.3)	0.9 (1.1)^∗^	1.2 (1.1)	0.5 (0.9)^∗^	0.7 (0.9)	0.4 (0.7)	0.4 (0.6)	0.2 (0.6)
Speech	2.2 (1.2)	1.6 (0.9)^∗^	1.6 (1.2)	0.9 (1.1)^∗^	1.1 (1.0)	0.6 (0.8)^∗^	0.7 (1.1)	0.3 (0.5)^∗^	0.5 (0.9)	0.1 (0.3)_∗_	0.3 (0.6)	0.1 (0.3)	0.1 (0.4)	0 (0)
General health	1.1 (1.3)	0.4 (0.6)^∗∗^	1.0 (1.4)	0.3 (0.6)^∗^	0.9 (1.4)	0.3 (0.7)^∗^	0.7 (1.2)	0.2 (0.6)	0.5 (1.0)	0.1 (0.3)^∗^	0.3 (0.7)	0.0 (0.2)^∗^	0.2 (0.5)	0.0 (0.2)^∗^
Comfort	2.5 (1.2)	1.6 (0.8)^∗∗^	2.1 (1.2)	1.3 (0.9)^∗∗^	1.8 (1.3)	1.1 (0.9)^∗^	1.5 (1.3)	0.7 (0.7)^∗∗^	1.2 (1.0)	0.6 (0.6)^∗∗^	0.9 (0.9)	0.4 (0.5)^∗∗^	0.7 (0.7)	0.4 (0.2)^∗∗^
Breath odor	1.6 (1.3)	1.63 (1.2)	1.6 (1.2)	1.3 (1.0)	1.2 (1.2)	0.9 (0.7)	1.0 (13)	0.4 (0.6)^∗^	0.9 (1.3)	0.3 (0.5)^∗^	0.8 (1.2)	0.1 (0.3)^∗∗^	0.7 (1.2)	0.1 (0.3)^∗∗^
Physical aspects	11.9 (5.2)	9.0 (3.6)^∗^	10.9 (5.0)	7.0 (3.8)^∗∗^	9.1 (5.5)	5.3 (4.2)^∗∗^	7.3 (5.7)	3.4 (3.0)^∗∗^	5.7 (5.1)	2.2 (2.2)^∗∗^	4.0 (4.0)	1.4 (1.4)^∗∗^	2.8 (3.1)	0.9 (1.2)^∗∗^
Social life	1.7 (1.3)	1.4 (1.0)	1.5 (1.3)	1.0 (0.9)	1.3 (1.2)	0.7 (0.8)	0.9 (1.2)	0.4 (0.6)	0.7 (1.1)	0.2 (0.4)^∗^	0.4 (0.8)	0.1 (0.4)	0.2 (0.6)	0.1 (0.3)
Romantic relationships	1.3 (1.4)	1.0 (1.2)	1.2 (1.3)	0.6 (0.9)	0.9 (1.0)	0.4 (0.8)	0.7 (1.0)	0.3 (0.6)^∗^	0.5 (0.9)	0.1 (0.3)^∗^	0.4 (0.7)	0.1 (0.3)^∗^	0.2 (0.5)	0.0 (0.2)
Smiling/laughing	2.2 (1.4)	1.4 (1.1)^∗^	2.0 (1.4)	1.2 (1.1)^∗^	1.8 (1.4)	1.0 (1.1)^∗^	1.3 (1.4)	0.6 (0.9)^∗^	1.0 (1.3)	0.3 (0.7)^∗∗^	0.5 (0.8)	0.2 (0.5)	0.3 (0.6)	0.1 (0.3)^∗^
Work/usual jobs	1.8 (1.4)	1.0 (1.1)^∗^	1.6 (1.4)	0.7 (0.8)^∗∗^	1.3 (1.4)	0.5 (0.7)^∗∗^	1.0 (1.4)	0.4 (0.6)^∗^	0.7 (1.1)	0.2 (0.4)^∗^	0.3 (0.7)	0.1 (0.3)^∗^	0.2 (0.4)	0.1 (0.3)
Finances	0.2 (0.7)	0.3 (0.7)	0.2 (0.6)	0.1 (0.3)	0.1 (0.4)	0.0 (0.2)	0.0 (0.2)	0.0 (0.2)	0.0 (0)	0.0 (0.2)	0.0 (0)	0.0 (0.2)	0.00 (0)	0.0 (0.2)
Social aspects	7.2 (5.0)	5.1 (4.2)	6.6 (5.0)	3.7 (3.4)^∗^	5.3 (4.5)	2.6 (3.0)^∗^	4.0 (4.5)	1.7 (2.3)^∗^	3.0 (4.0)	0.8 (1.4)^∗∗^	1.7 (2.7)	0.5 (0.9)^∗^	0.9 (1.9)	0.3 (0.7)
Confidence	0.7 (1.2)	0.4 (0.7)	0.7 (1.2)	0.2 (0.5)^∗^	0.6 (1.0)	0.1 (0.4)^∗∗^	0.4 (0.8)	0.1 (0.4)^∗^	0.3 (0.7)	0.0 (0.2)	0.2 (0.5)	0 (0)	0.1 (0.4)	0 (0)
Carefree manner	1.8 (1.6)	1.3 (1.1)	1.7 (1.5)	0.9 (0.9)^∗^	1.5 (1.5)	0.7 (0.9)^∗^	1.2 (1.4)	0.4 (0.7)^∗^	0.9 (1.3)	0.3 (0.5)^∗^	0.6 (0.9)	0.1 (0.4)^∗^	0.3 (0.6)	0.1 (0.3)
Sleep/ability to relax	1.7 (1.5)	0.9 (0.9)^∗^	1.6 (1.5)	0.5 (0.9)^∗∗^	1.2 (1.4)	0.5 (0.8)^∗^	0.9 (0.3)	0.3 (0.6)^∗^	0.7 (1.1)	0.2 (0.4)^∗^	0.5 (0.8)	0.0 (0.2)^∗∗^	0.3 (0.5)	0.0 (0.2)^∗^
Moon	1.7 (1.5)	0.8 (0.9)^∗∗^	1.5 (1.4)	0.6 (0.9)^∗∗^	1.1 (1.4)	0.5 (0.9)^∗^	0.9 (1.4)	0.3 (0.7)^∗^	0.7 (1.2)	0.2 (0.5)^∗^	0.4 (0.8)	0.1 (0.3)^∗^	0.3 (0.5)	0.1 (0.3)
Personality	1.0 (1.4)	0.4 (0.7)^∗^	0.8 (1.3)	0.3 (0.7)	0.8 (1.4)	0.0 (0.6)^∗^	0.7 (0.2)	0.1 (0.3)^∗^	0.6 (1.1)	0.1 (0.3)^∗^	0.3 (0.7)	0.0 (0.2)^∗^	0.2 (0.5)	0 (0)^∗^
Psychological aspects	7.0 (6.0)	3.7 (3.1)^∗^	6.3 (5.7)	2.5 (3.1)^∗∗^	5.3 (5.5)	1.9 (2.9)^∗∗^	4.2 (5.2)	1.3 (2.0)^∗∗^	3.2 (4.6)	0.7 (1.3)^∗∗^	2.0 (2.9)	0.3 (0.7)^∗∗^	1.2 (2.1)	0.2 (0.5)^∗^
Total QOL	26.1 (15.4)	17.9 (9.9)^∗^	23.8 (14.9)	13.3 (9.3)^∗∗^	19.7 (14.9)	9.8 (9.4)^∗∗^	15.5 (15.1)	6.4 (6.8)^∗∗^	11.8 (13.3)	3.8 (4.3)^∗∗^	7.7 (9.2)	2.2 (2.4)^∗∗^	4.9 (6.6)	1.3 (1.9)^∗∗^

^∗^ *p* < 0.05*t*-test between groups. ^∗∗^*p* < 0.01*t*-test between groups.

## Data Availability

The data used to support the findings of this study are included within the article.

## References

[1] Şimşek Kaya G., Yapıcı Yavuz G., Saruhan N. (2019). The influence of flap design on sequelae and quality of life following surgical removal of impacted mandibular third molars: a split-mouth randomised clinical trial. *Journal of Oral Rehabilitation*.

[2] Duarte-Rodrigues L., Miranda E. F. P., Souza T. O., de Paiva H. N., Falci S. G. M., Galvão E. L. (2018). Third molar removal and its impact on quality of life: systematic review and meta-analysis. *Quality of Life Research*.

[3] Daniels S. E., Bandy D. P., Christensen S. E. (2011). Evaluation of the dose range of etoricoxib in an acute pain setting using the postoperative dental pain model. *The Clinical Journal of Pain*.

[4] Albuquerque A. F. M., Fonteles C. S. R., do Val D. R. (2017). Effect of pre-emptive analgesia on clinical parameters and tissue levels of TNF-*α* and IL-1*β* in third molar surgery: a triple-blind, randomized, placebo- controlled study. *International Journal of Oral and Maxillofacial Surgery*.

[5] Costa F. W. G., Soares E. C. S., Esses D. F. S. (2015). A split-mouth, randomized, triple-blind, placebo-controlled study to analyze the pre-emptive effect of etoricoxib 120 mg on inflammatory events following removal of unerupted mandibular third molars. *International Journal of Oral and Maxillofacial Surgery*.

[6] Malmstrom K., Sapre A., Couglin H., Agrawal N. G., Mazenko R. S., Fricke J. R. (2004). Etoricoxib in acute pain associated with dental surgery: a randomized, double- blind, placebo- and active comparator-controlled dose-ranging study. *Clinical Therapeutics*.

[7] Malmstrom K., Ang J., Fricke J. R., Shingo S., Reicin A. (2004). The analgesic effect of etoricoxib relative to that of acetaminophen analgesics: a randomized, controlled single-dose study in acute dental impaction pain. *Current Medical Research and Opinion*.

[8] Woolf C. J., Chong M. S. (1993). Preemptive analgesia—treating postoperative pain by preventing the establishment of central sensitization. *Anesthesia and Analgesia*.

[9] Viscusi E. R., Frenkl T. L., Hartrick C. T. (2012). Perioperative use of etoricoxib reduces pain and opioid side-effects after total abdominal hysterectomy: a double-blind, randomized, placebo-controlled phase III study. *Current Medical Research and Opinion*.

[10] Srivastava S., Gupta D., Naz A., Rizvi M. M., Singh P. K. (2012). Effects of preoperative single dose etoricoxib on postoperative pain and sleep after lumbar diskectomy: prospective randomized double blind controlled study. *Middle East Journal of Anaesthesiology*.

[11] Somri M., Hawash N., Hadjittofi C. (2017). Protective multimodal analgesia with etoricoxib and spinal anesthesia in inguinal hernia repair: a randomized controlled trial. *Journal of Anesthesia*.

[12] Isola G., Matarese M., Ramaglia L., Cicciù M., Matarese G. (2019). Evaluation of the efficacy of celecoxib and ibuprofen on postoperative pain, swelling, and mouth opening after surgical removal of impacted third molars: a randomized, controlled clinical trial. *International Journal of Oral and Maxillofacial Surgery*.

[13] Riendeau D., Percival M. D., Brideau C. (2001). Etoricoxib (MK-0663): preclinical profile and comparison with other agents that selectively inhibit cyclooxygenase-2. *The Journal of Pharmacology and Experimental Therapeutics*.

[14] Negreiros R. M., Biazevic M. G., Jorge W. A., Michel-Crosato E. (2012). Relationship between oral health-related quality of life and the position of the lower third molar: postoperative follow-up. *Journal of Oral and Maxillofacial Surgery*.

[15] World Health Organization (2001). *International Classification of Functioning, Disability and Health*.

[16] Mcgrath C., Bedi R. (2001). An evaluation of a new measure of oral health related quality of life--OHQoL-UK(W). *Community Dental Health*.

[17] Patrignani P., Capone M. L., Tacconelli S. (2003). Clinical pharmacology of etoricoxib: a novel selective COX-2 inhibitor. *Expert Opinion on Pharmacotherapy*.

[18] Warner T. D., Mitchell J. A. (2004). Cyclooxygenases: new forms, new inhibitors, and lessons from the clinic. *The FASEB Journal*.

[19] Cochrane D. J., Jarvis B., Keating G. M. (2002). Etoricoxib. *Drugs*.

[20] Majid O. W. (2011). Submucosal dexamethasone injection improves quality of life measures after third molar surgery: a comparative study. *Journal of Oral and Maxillofacial Surgery*.

[21] Batinjan G., Zore Z., Čelebić A., Papić M., Gabrić Pandurić D., Filipović Zore I. (2014). Thermographic monitoring of wound healing and oral health-related quality of life in patients treated with laser (aPDT) after impacted mandibular third molar removal. *International Journal of Oral and Maxillofacial Surgery*.

[22] Ramos-Remus C. R., Hunsche E., Mavros P., Querol J., Suarez R. (2004). Evaluation of quality of life following treatment with etoricoxib in patients with arthritis or low-back pain: an open label, uncontrolled pilot study in Mexico. *Current Medical Research and Opinion*.

[23] Eichler H. G., Mavros P., Geling O., Hunsche E., Kong S. (2005). Association between health-related quality of life and clinical efficacy endpoints in rheumatoid arthritis patients after four weeks treatment with anti-inflammatory agents. *International Journal of Clinical Pharmacology and Therapeutics*.

[24] Majid O. W., Al-Mashhadani B. A. (2014). Perioperative bromelain reduces pain and swelling and improves quality of life measures after mandibular third molar surgery: a randomized, double-blind, placebo-controlled clinical trial. *Journal of Oral and Maxillofacial Surgery*.

[25] Shugars D. A., Gentile M. A., Ahmad N. (2006). Assessment of oral health-related quality of life before and after third molar surgery. *Journal of Oral and Maxillofacial Surgery*.

[26] Sancho-Puchades M., Valmaseda-Castellón E., Berini-Aytés L., Gay-Escoda C. (2012). Quality of life following third molar removal under conscious sedation. *Medicina Oral, Patología Oral y Cirugía Bucal*.

[27] Savin J., Ogden G. R. (1997). Third molar surgery--a preliminary report on aspects affecting quality of life in the early postoperative period. *The British Journal of Oral & Maxillofacial Surgery*.

[28] Colorado-Bonnin M., Valmaseda-Castellón E., Berini-Aytés L., Gay-Escoda C. (2006). Quality of life following lower third molar removal. *International Journal of Oral and Maxillofacial Surgery*.

[29] Ibikunle A. A., Adeyemo W. L., Ladeinde A. L. (2016). Oral health-related quality of life following third molar surgery with either oral administration or submucosal injection of prednisolone. *Oral and Maxillofacial Surgery*.

[30] Isola G., Matarese M., Ramaglia L., Iorio-Siciliano V., Cordasco G., Matarese G. (2019). Efficacy of a drug composed of herbal extracts on postoperative discomfort after surgical removal of impacted mandibular third molar: a randomized, triple-blind, controlled clinical trial. *Clinical Oral Investigations*.

[31] Renner B., Walter G., Strauss J., Fromm M. F., Zacher J., Brune K. (2012). Preoperative administration of etoricoxib in patients undergoing hip replacement causes inhibition of inflammatory mediators and pain relief. *Eur J Pain*.

[32] Beech A. N., Haworth S., Knepil G. J. (2018). Effect of a domiciliary facial cooling system on generic quality of life after removal of mandibular third molars. *The British Journal of Oral & Maxillofacial Surgery*.

[33] Sáez-Alcaide L. M., Molinero-Mourelle P., González-Serrano J., Rubio-Alonso L., Bornstein M. M., López-Quiles J. (2020). Efficacy of a topical gel containing chitosan, chlorhexidine, allantoin and dexpanthenol for pain and inflammation control after third molar surgery: a randomized and placebo-controlled clinical trial. *Medicina oral, patologia oral y cirugia bucal*.

[34] Hakeberg M., Cunha L. (2009). Dental anxiety and pain related to dental hygienist treatment. *Acta Odontologica Scandinavica*.

